# Foodborne Transmission of Hepatitis E Virus from Raw Pork Liver Sausage, France

**DOI:** 10.3201/eid2011.140791

**Published:** 2014-11

**Authors:** Christophe Renou, Anne-Marie Roque Afonso, Nicole Pavio

**Affiliations:** Hôpital d’Hyères, Hyères, France (C. Renou);; Hôpital Paul Brousse, Virologie, Villejuif, France (A.-M. Roque Afonso);; French Agency for Food, Environmental and Occupational Health and Safety, Maisons-Alfort, France (N. Pavio)

**Keywords:** HEV, transmission, foodborne, viruses, France, pork, zoonoses

**To the Editor:** The number of sporadic autochthonous cases of acute hepatitis E is increasing in many industrialized countries (*1*). These cases involve hepatitis E virus (HEV) genotypes 3 and 4, which are zoonotic. Although risk for foodborne transmission from pork is now recognized, we report here direct HEV transmission through ingestion of raw pig liver sausages (figatellu [plural: figatelli]) in southeastern France.

The index case-patient was a 45-year-old woman from Hyères (southeastern France) who had no underlying medical condition. She visited her general practitioner on December 17, 2013, reporting 3 days of weakness. Acute hepatitis was diagnosed 2 days later on the basis of elevated liver enzymes (alanine aminotransferase 1,265 IU/L [reference <35 IU/L]) and bilirubin (65 μmol/L [reference <17 μmol/L]). Serum markers for acute hepatitis A, B, and C; cytomegalovirus; and Epstein-Barr virus were negative. Jaundice appeared on December 19, and the patient was referred to the Medical Unit of Hyères for additional investigations. A serum sample collected on December 20 tested positive for HEV RNA; viral load was 3.3 log_10_ IU/mL (Ceeram, La Chapelle sur Erdre, France), and IgM and IgG against HEV were found (Wantai, Beijing, China), which led to the diagnosis of acute hepatitis E. The HEV genotype was 3f, as determined from the phylogenetic analysis of a portion of the open reading frame (ORF) 2 (*2*). The index case-patient recovered by the end of January; HEV viremia was undetectable on January 17, 2014.

The index case-patient and her family regularly ate figatelli (raw pork liver sausages) made in Corsica. The patient had most recently eaten figatelli at a lunch with 8 family members on October 28, 2013, seven weeks before illness onset. After receiving informed consent, we conducted laboratory investigations of samples from the other family members; tests included HEV serology and HEV RNA detection in serum and fecal samples. Samples were obtained from family members during January 8–21, 2014 (41–54 days after the lunch). Positive HEV IgM and detectable HEV RNA were found in the serum of the index case-patient’s daughter, who was asymptomatic. Because the sample was tested 10 weeks after the family lunch, the daughter’s HEV viral load was too low to enable sequence characterization and clustering of HEV strains. Three other family members were IgG positive for HEV, indicating previous HEV infection. Leftover sausages had been kept frozen and were available for HEV testing.

HEV RNA was detectable from the leftover sausages, and HEV sequences were amplified in 2 different genomic regions (ORF1: RNA-dependent RNA polymerase and ORF2), as described previously (*2*). Comparison with the index case-patient’s sequences showed 100% nt identity for both regions (Figure). Samples of food and samples from the index case-patient were analyzed in 2 independent laboratories to avoid any cross-contamination. The level of contamination of the figatellu was ≈4.8 10^4^ copies of HEV RNA/g of sausage (*3*).

**Figure:**
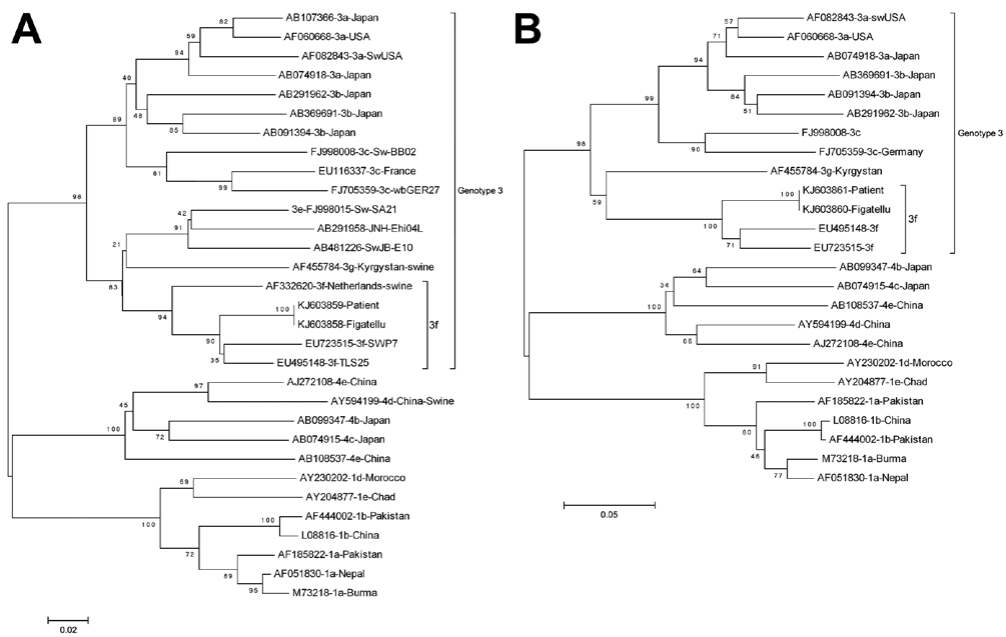
Phylogenetic analysis of partial open reading frame (ORF) 2 and ORF1 sequences of hepatitis E virus (HEV). Phylogenetic trees were constructed in MEGA6 software (http://www.megasoftware.net) by using the neighbor-joining method from a Kimura 2-parameter distance matrix based on partial nucleotide sequences of ORF2 (A) and ORF1 (B). Bootstrap values obtained from 500 resamplings are shown. Sequences were retrieved from the serum of a 45-year-old woman in France in whom hepatitis E was diagnosed in December 2013 and from frozen leftovers of the figatellu she had eaten in October 2013. Sequences obtained from the figatellu (GenBank accession nos. KJ603858 and KJ603860) were 100% nt identical to sequences obtained from the index case-patient (GenBank accession nos. KJ603859 and KJ603861). Reference sequences are indicated by their GenBank accession numbers. Scale bars indicate nucleotide substitutions per site.

Figatellu, a dried sausage, contains 30% pork liver and no heating step occurs during its manufacture. Usually deep cooking is recommended on the package, but consumers might not follow the cooking recommendation; also, figatelli can be sold in small local shops with no label. In the instance reported here, the figatellu was sold without any warning label and was eaten raw.

That HEV was transmitted through ingestion of contaminated food is supported by the following evidence. First, 3 case reports have provided direct evidence of HEV transmission through ingestion of contaminated animal food products with identical or near identical sequences between the patients and the contaminated food they ate. Two cases occurred in the early 2000s in Japan through consumption of grilled wild boar (*4*) or sashimi of Sika deer (*5*); the third, reported recently in Spain, was transmitted through ingestion of pig meat (*6*). Second, HEV widely infects domestic pigs and wild boar (*7*). Third, swine and human HEV strains have genetic similarities and, in some cases, are indistinguishable (*1*). Fourth, in Marseille, France, a case–control study identified ingestion of figatellu as a risk factor for HEV infection. Genetic similarities were found between sequences isolated from patients with autochthonous hepatitis E and nonrelated figatelli purchased in the same region (*8*). Finally, infectious virus, replicating in a 3-dimensional culture system, was identified in a HEV RNA-positive figatellu (*9*).

In the present study, the homology between sequences recovered from the index case-patient and those recovered from leftovers of figatellu provides additional proof of HEV foodborne transmission in a Western country. In France, information about the risk for HEV transmission through the ingestion of such delicatessen was published by French authorities in 2010 (*10*), but the present case demonstrates that public education and warning, or larger and more explicit labels on the package, must be improved to reduce the risk for HEV exposure.

## References

[1] Dalton HR, Bendall R, Ijaz S, Banks M. Hepatitis E: an emerging infection in developed countries. Lancet Infect Dis. 2008;8:698–709. 10.1016/S1473-3099(08)70255-X 18992406

[2] Haïm-Boukobza S, Ferey MP, Vétillard AL, Jeblaoui A, Pélissier E, Pelletier G, Transfusion-transmitted hepatitis E in a misleading context of autoimmunity and drug-induced toxicity. J Hepatol. 2012;57:1374–8. 10.1016/j.jhep.2012.08.001 22885386

[3] Barnaud E, Rogee S, Garry P, Rose N, Pavio N. Thermal inactivation of infectious hepatitis E virus in experimentally contaminated food. Appl Environ Microbiol. 2012;78:5153–9. 10.1128/AEM.00436-12 22610436 PMC3416424

[4] Li TC, Chijiwa K, Sera N, Ishibashi T, Etoh Y, Shinohara Y, Hepatitis E virus transmission from wild boar meat. Emerg Infect Dis. 2005;11:1958–60. 10.3201/eid1112.051041 16485490 PMC3367655

[5] Tei S, Kitajima N, Takahashi K, Mishiro S. Zoonotic transmission of hepatitis E virus from deer to human beings. Lancet. 2003;362:371–3. 10.1016/S0140-6736(03)14025-1 12907011

[6] Riveiro-Barciela M, Minguez B, Girones R, Rodriguez-Frias F, Quer J, Buti M. Phylogenetic demonstration of hepatitis E infection transmitted by pork meat ingestion. J Clin Gastroenterol. 2014; Epub ahead of print. 10.1097/MCG.0000000000000113 24637729

[7] Pavio N, Meng XJ, Renou C. Zoonotic hepatitis E: animal reservoirs and emerging risks. Vet Res. 2010;41:46. 10.1051/vetres/2010018 20359452 PMC2865210

[8] Colson P, Borentain P, Queyriaux B, Kaba M, Moal V, Gallian P, Pig liver sausage as a source of hepatitis E virus transmission to humans. J Infect Dis. 2010;202:825–34. 10.1086/655898 20695796

[9] Berto A, Grierson S, Hakze-van der Honing R, Martelli F, Johne R, Reetz J, Hepatitis E virus in pork liver sausage, France. Emerg Infect Dis. 2013;19:264–6 . 10.3201/eid1902.121255 23347828 PMC3563277

[10] Ministère du Travail, de l’Emploi. et de la Santé. Prévenir l’hépatite E chez les personnes susceptibles de développer une forme grave [cited 2014 Sep 17]. http://www.sante.gouv.fr/IMG/pdf/Fiche_Hepatite_E.pdf

