# Correction: Vol. 20, No. 11

**DOI:** 10.3201/eid2102.C12102

**Published:** 2015-02

**Keywords:** errata, erratum, correction

The name of author Anne-Marie Roque-Afonso was listed incorrectly in the article Foodborne Transmission of Hepatitis E Virus from Raw Pork Liver Sausage, France (C. Renouet al.). The article has been corrected online (http://wwwnc.cdc.gov/eid/article/20/11/14-0791_article.htm).

